# Performance of Distress Thermometer and Associated Factors of Psychological Distress among Chinese Cancer Patients

**DOI:** 10.1155/2020/3293589

**Published:** 2020-09-22

**Authors:** Sudip Thapa, Huihui Sun, Gaurab Pokhrel, Bangyan Wang, Sanuja Dahal, Shiying Yu

**Affiliations:** ^1^Cancer Center of Tongji Hospital, Tongji Medical College, Huazhong University of Science and Technology, Wuhan 430030, China; ^2^Department of Urology, Tongji Hospital, Tongji Medical College, Huazhong University of Science and Technology, Wuhan 430030, China; ^3^School of Nursing, Tongji Medical College, Huazhong University of Science and Technology, Wuhan 430030, China

## Abstract

**Objective:**

We aimed to examine the performance of the distress thermometer (DT) and identify the prevalence and risk factors associated with psychological distress (PD) in heterogeneous cancer patients.

**Methods:**

This cross-sectional study enrolled 1496 heterogeneous cancer patients from the inpatient and outpatient departments. Receiver operating characteristic analysis (ROC) of DT was evaluated against the Hospital Anxiety and Depression Scale-Total (HADS-T ≥15). An area under the curve (AUC), sensitivity, specificity, positive predictive value (PPV), negative predictive value (NPV), and clinical utility index were calculated. Multiple binary logistic regression was used to identify the factors associated with PD.

**Results:**

Referring to ROC analysis, DT showed good discriminating accuracy (AUC = 0.88). A cutoff score of 4 was established, and it yielded sensitivity (0.81), specificity (0.88), PPV (0.87), NPV (0.82), and clinical utility indexes (screening utility = 0.71 and case-finding utility = 0.73). 46.5% of our participants was distressed. Lower education levels (odd ratio (OR) = 1.39), advanced stage (OR = 1.85), active disease status (OR = 1.82), lack of exercise (OR = 3.03), diagnosis known (OR = 0.64), emotional problems (OR = 3.54), and physical problems (OR = 8.62) were the predictive factors for PD.

**Conclusion:**

DT with a cutoff score (≥4) is a comprehensive, appropriate, and practical initial screener for PD in cancer patients. Predicting factors should be considered together for effective management of PD in such population.

## 1. Introduction

Anxiety and depression are common after a cancer diagnosis. Though anxiety and depression are different clinical entities, they are generally referred to as psychological distress (PD). Cancer-related PD has profound negative impacts on patients' health as it is associated with poor quality of life [1], poor satisfaction with medical treatments [2], and suicidal ideation [3]. Timely identification and treatment of PD are beneficial in reducing its negative consequences among cancer patients [4]. Therefore, the National Comprehensive Cancer Network (NCCN) Guidelines for Distress Management recommend that every cancer patient should be screened for PD and managed accordingly [5].

Screening is defined as the presumptive identification of unrecognized disease. Screening tool is an initial investigation of a disease or condition. There are various screening tools for PD such as the Hospital Anxiety and Depression Scale (HADS), Brief Symptom Inventory-18 (BSI-18), Symptom Checklist-90, and other psychiatric interview tools. These tools are lengthy, time-consuming, and bothersome for patients to complete. Therefore, the NCCN Distress Management Panel and available studies recommend the use of the distress thermometer (DT) as a screening tool for PD. The DT is ultrashort, easy to use, and nonstigmatizing for the patients [5–7]. DT is valid in many countries, and its recommended cutoff score is 4 [8, 9]. However, its area under the ROC curve (AUC = 0.47–0.91), sensitivity (SE = 0.42–1.00), specificity (SP = 0.36–0.98), positive predictive value (PPV = 0.23–0.95), negative predictive value (NPV = 0.68–0.97), and the optimal cutoff score (3–7) vary among different countries, studies, clinical settings, cancer types, and patients' sociodemographic characteristics [8–15].

In China, cancer is a common public health burden, and caring cancer patients is commonly troublesome by nondisclosure of the diagnosis [16]. Even patients who knew their diagnosis often keep a secret because they are influenced by their philosophical and medical beliefs [17]. This could lead to inaccurate expressions of their feelings about the diagnosis and the level of their perceived distress. Over the past decades, education and health care facilities in China have improved significantly. These improvements have brought knowledge, awareness, and acceptance of cancer. Interestingly, a recent study has shown that there is an increasing trend in the disclosing of diagnosis in Chinese cancer patients [18]. Thus, the significance of validating DT in recent time remains very important. DT performance in China differs with geographic locations from poor to excellent, and its cutoff scores vary between 3 and 5 [11, 12, 19–22]. To date, most of the Mainland Chinese studies only measured the accuracy of DT against specific cancer types. Available studies (Table 1) are limited by clinical settings (inpatient) and insufficient study population that may not effectively represent the overall cancer population [12, 20–24]. This raises the question as to whether one comprehensive optimal cutoff score to distinguish PD in general cancer patients regardless of the clinical setting (inpatient and outpatient) and cancer types is needed. Thus, this study aimed to (1) evaluate the performance and determine a comprehensive DT cutoff score to measure PD in heterogeneous cancer patients and (2) investigate the prevalence and factors associated with PD in these populations.

## 2. Methods

### 2.1. Participants

This cross-sectional study was conducted from July 2018 to January 2019 in the inpatient and outpatient departments of the Cancer Center of Tongji Hospital, Tongji Medical College, Huazhong University of Science and Technology. The inclusion criteria included: age >18 years, literate with normal cognitive functions, diagnosed with cancer, and willing to participate. Informed consent was obtained from the eligible participants and were requested to complete the questionnaire by themselves or with the help of a research assistant. Patients with a history of psychiatric illness were excluded from this study. A questionnaire including sociodemographic information such as age, sex, marital status, education level, occupation, monthly income, aware/unaware of diagnosis, and exercise habit was distributed to each participant. Exercise habit was categorized into 2 groups: frequent exercise (>150 min/week) and lack of exercise (<150 min/week). Clinical information such as disease status, duration, stage and type of cancer, and treatment received were collected from the computer records.

This study was a part of a clinical trial which was approved by the Research Ethical Board of Tongji Medical College (CFDA#2015R006398), and the Declaration of Helsinki was strictly followed.

### 2.2. Measurements

DT is a single item, self-reported, thermometer-shaped visual analog scale consisting of 11 points ranging from 0 (no distress) to 10 (extreme distress) that measures PD over the past 7 days. The problem list (PL) recommended by NCCN is used to identify the nature of the possible problems that cause PD. It contains a list of 36 possible problems that are categorized in 5 domains (practical, family, emotional, physical problems, and spiritual or religious concerns), but the Chinese version of PL contains 40 possible problems. DT accompanied with PL is a validated tool in China [19].

HADS is widely used for defining the presence of cancer-specific mood disorders. HADS is a 14 item self-rated screening tool. It includes anxiety (HADS-A) and depression (HADS-D) subscales, and each subscale contains 7 items [25]. Clinically, the two subscales of HADS (HADS-A and HADS-D) considered a single measurement of HADS-T (PD). As a screening tool, HADS has good accuracy and validity [26]. Most of the studies have used HADS as a standard criterion for validating DT [8, 9], and many studies have considered HADS-T ≥15 as a cutoff score for PD [8, 9, 15, 22].

### 2.3. Statistical Analysis

The receiver operating characteristic (ROC) curve analysis was performed to identify the cutoff score of DT against the HADS-T ≥15. Youden index (i.e., sensitivity + specificity-1) was calculated, and its largest value determines the optimal cutoff score. The AUC was used to measure the overall discriminative accuracy of DT against the HADS-T, and the value of 0.5–0.7, 0.7–0.9, and ≥0.9–1.0 reflected low, moderate, and excellent discriminative accuracy, respectively.

The SE, SP, PPV, and NPV were evaluated at each DT cutoff score against the HADS-T. Interpretation of screening tests can be improved by clinical utility index (UI) as it gives more information about the performance of the screening tool [27] and was also applied in our study. SE and SP are measures of occurrence, while PPV and NPV are measures of discrimination. The positive utility index (UI+) = SE × PPV provides rule-in accuracy (case-finding), and negative utility index (UI−) = SP × NPV provides rule-out accuracy (screening) of the test [27]. Furthermore, the main factors contributing to PD was identified using multiple binary logistic regression model analysis. The statistical package for social science (SPSS, version 20.0, Chicago, IL, USA) was used to analyze the data, and a value of *P* ≤ 0.05 was considered to be statistically significant.

## 3. Results

### 3.1. Demographic and Clinical Characteristics

A total of 1518 patients were approached, 22 (1.5%) denied their participation, and 1496 (98.5% response rate) were included in the final analysis, of which 784 were inpatient and 712 outpatient. The mean age of patients was 54.4 ± 11.2 years. Among the total participants, 51.3% was female, 56.6% attended junior high school or below education, and 67.5% lacked regular exercise. Regarding clinical characteristics, 80.7% had noncomplete remission (active disease status), 89.9% knew their diagnosis, 38.5% had stage IV cancer, 55.7% received combined treatment, 33.6% had lung cancer, and followed by digestive cancer 27.7% (Table 2).

### 3.2. Descriptive Statistics of Psychological Distress on Problem List Domains

The mean score of DT was 3.3 ± 2.6. At DT (≥4), 46.5% (*n* = 696) of participants was found to be distressed. The reported major sources of distress were physical problems (*n* = 1174, 78.5%) followed by emotional problems (*n* = 1064, 71.1%), practical problems (*n* = 894, 59.8%), family problems (*n* = 464, 31.0%), and spiritual problems (*n* = 12, 0.8%) (Table 2).

### 3.3. ROC Analysis and Optimal Cutoff Score

**Figure 1:**
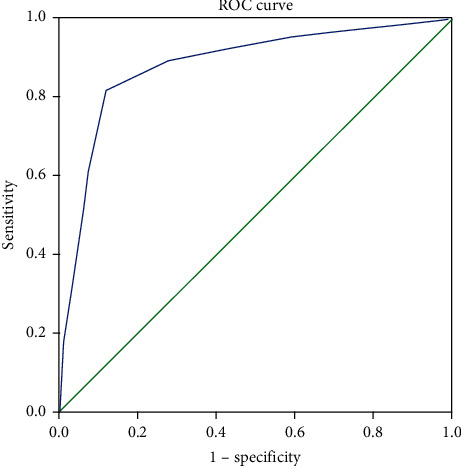
Receiver operating characteristic (ROC) curve of the distress thermometer (DT) score against the Hospital Anxiety and Depression Scales-Total (HADS-T) cutoff score. The area under the ROC curve (AUC) was 0.88.

DT showed a good discriminating accuracy (AUC = 0.88, 95% CI 0.865–0.901) between distress and nondistress against HADS-T ≥15. A cutoff score of 4 on DT correctly identified 0.81 of HADS distress cases (SE) and 0.88 of HADS nondistress cases (SP) with PPV (i.e., proportions of detecting true positives) and NPV (i.e., proportions of detecting true negative) of 0.87 and 0.82, respectively. The UI calculation demonstrated that DT had good accuracy in both screening (UI+ = 0.71) and case-finding (UI− = 0.73) (Figure 1 and Table 3).

### 3.4. Factors Influencing Distress

Multiple binary logistic regression analysis revealed that lower education levels (OR = 1.39, *P*=0.01, 95% CI 1.060–1.825), active disease status (OR = 1.82, *P*=0.001, 95% CI 1.274–2.619), advanced cancer stage (OR = 1.85, *P* < 0.001, 95% CI 1.424–2.405), lack of exercise (OR = 3.03, *P* < 0.001, 95% CI 2.307–3.989), emotional problems (OR = 3.54, *P* < 0.001, 95% CI 2.540–4.942), and physical problems (OR = 8.62, *P* < 0.01, 95% CI 5.468–13.594) were the risk factors for PD. Whereas patients who knew their diagnosis (OR = 0.64, *P*=0.02, 95% CI 0.435–0.951) were not likely to be at risk of PD (Table 4).

## 4. Discussion

In cancer care, PD is considered as the “Sixth Vital sign” [28]. A generalized, efficient, and accurate screening tool is needed for timely identification and proper management of distressed patients. To the best of our knowledge, this is the first study conducted in China to examine the performance of the DT including both inpatient and outpatient among heterogeneous cancer population.

We found that the DT demonstrated good discriminating accuracy (AUC = 0.88) against HADS-T, which showed that the DT is an easier and acceptable tool. This finding is in agreement with earlier studies, where AUC ranged from 0.47 to 0.91 [8–12]. Against the HADS-T (≥15), an optimal cutoff score of 4 was identified on the DT. This result was exactly in line with the current NCCN guidelines [5] and confirmed by other existing studies from different cultural backgrounds performed in Saudi Arabia [29], Korea [2], Italy [6], America [7], and China [19, 21]. Our result is also consistent with a study conducted in different clinical settings such as inpatient [24], outpatient [6, 7], and inpatient and outpatient combined [29] in heterogeneous and homogeneous cancer types [8, 9, 29]. Collectively, it is possible to infer that the cutoff score may not be influenced by geography, cultural issues, and screening settings but may depend on the disease “cancer” itself. These findings provide more evidence in generalizing the DT cutoff score across different cancer populations in different clinical settings.

However, some studies found higher cutoff scores in outpatient [1], lymphoma, [12], intracranial tumor, [14], shortly after breast cancer diagnosis [15], and advanced cancer inpatient with pain [22]. It is difficult to explain the exact cause for differences in cutoff scores between studies. Perception of PD can depend on an individual patient, which may differ with the course of cancer illness, treatment, and its specific physical problems. The DT has been translated from the English language, and there is no universal translating instrument that can exactly substitute the word “distress.” Additionally, different translated phrases have yielded different cutoff scores in the same region of study [11]. There is no common method for selecting the cutoff score among the studies. All these factors may contribute for varying cutoff scores.

A cutoff score of 4 correctly detected 0.81 of distress cases (SE) and 0.88 of nondistress cases (SP). Among the previous studies, DT's sensitivity ranged from poor to excellent (0.42–1.00) [8, 20]. The SE of this study was comparable to meta-analysis by Ma et al. (0.82) [9] and studies conducted in different geographical location including China (0.82 and 0.86) [11, 22], Korea (0.83) [2], and Spain (0.90) [13]. Regarding specificity, its value ranged from 0.36 to 0.98 and was comparable with the majority of previous studies [2, 8, 11–13, 15, 22, 29]. Higher SE and SP of the DT can efficiently recognize a large proportion of patients with or without PD. The consistency of our result with available studies may be due to DT's nonstigmatizing nature and its unique thermometer-shaped visual analog scale. This makes it easier for the patients to choose the appropriate score corresponding to their perceived PD level.

We obtained a PPV of 0.87 and NPV of 0.82 at a cutoff score of 4, resulting in fewer false-positives (13%) and false-negative (18%) rates. These are notable findings, which were not evaluated in most of the previous Chinese studies. Our results are comparable to studies performed across different countries, where PPV and NPV ranged from 0.35 to 95.8 and 0.68 to 97, respectively [8, 15, 22, 29]. These were important findings, which can prevent both over-misdiagnosis and underdiagnosis, reducing unnecessary patient volume, investigation, overtreatment, and financial burden. More importantly, UI+ (0.71) and UI− (0.73) also demonstrated that the DT had good accuracy in both ruling-in and ruling-out PD cases indicating that the DT has a good screening performance. Interestingly, the UI in our study was superior to Martinez et al. study [13]. The plausible cause for these discrepancies may be due to study methodology (i.e., BSI-18 vs. HADS) and differences in the characteristics of the included population.

The reported prevalence of distress in cancer patients around the globe ranges from 22.1% to 89.1% [3, 13, 29–31]. In our study, the incidence of PD was 46.5%. Together with these results, we can infer that PD is a serious global problem among different cancer patients that needs routine monitoring, and providing appropriate management for PD is an urgent need.

Consistent with previous studies, sociodemographic factors such as lower education levels and advanced cancer stage were independently associated with PD in the present analysis [29, 32]. The well-educated populations are socially engaged and live healthier and longer because they have higher cognitive skills such as reading, writing, thinking, reasoning, problem solving, and possess strong decision power [33]. Whereas populations with lower educational backgrounds might have low socioeconomic status and limited access to available resources. All these factors may add stress in life causing PD. Diaz-Frutos et al. found that advanced or terminal stage patients were hopeless and emotionally disturbed [3]. Moreover, the possible cause for PD might be the thought of death, which is fear-provoking and painful. Also, in this study, patients with active disease status were at a greater risk of PD. During the active disease, patients may receive a series of anticancer treatments (surgery, chemotherapy, radiation-therapy, and biological therapy). These have several adverse effects, which might increase PD. Hence, while screening, healthcare professionals should prioritize vulnerable patients groups such as lower education levels, advanced cancer stage, and active disease status, which could be helpful for PD prevention.

Patients who know their diagnosis have less distress and a better quality of life [34, 35]. We noted a similar finding where patients knowing their diagnosis were less likely to be at risk of PD. Nondisclosure of the diagnosis is the most common problem in cancer caring [16]. Disclosure may help patient to understanding the true situation of the disease that might lower the disbelief and hopelessness about their disease conditions. Awareness of diagnosis may enable them to cope with their true conditions and in turn improve patient satisfaction with care. The appropriate approach of disclosure could be a valuable support in cancer management.

Patients with a lack of physical exercise are strongly associated with higher PD [36]. The randomized controlled trial by Chen et al. mentioned that exercise habits are beneficial in relieving anxiety and depression [37]. Major health organization recommend that cancer patients should have at least 150 min of moderate-intensity or 75 min of high-intensity exercise combined with a minimum of two strengthening exercises sessions weekly [38]. Our study also demonstrated that lack of exercise was a risk factor for PD. Thus, exercise can be considered as a natural, safe, cost-effective, and nonpharmacological alternative treatment to reduce cancer-specific PD. Therefore, regular exercise might be worth recommending.

In our study, emotional and physical problems of the PL were strongly associated (exhibited high odd ratios and highly significant *P* value) with PD. This was consistent within Chinese studies (i.e., lymphoma and advanced cancer with pain) and a Saudi Arabic study in mixed cancer patients [12, 22, 29]. This finding adds further evidence that emotional and physical problems may be independent of the ethnic group. Factors such as age, gender, marital status, occupation, time since diagnosis, cancer type, and treatment were not associated with distress and are consistent with previous studies [22, 29].

Despite the higher response rates and larger population size from two different clinical settings (inpatient and outpatient), our study had some limitations. First, there exists a possible selection bias because this was a single center cross-sectional study. Also, regarding DT validation, we only selected the HADS criteria, and diagnostic interviews were not performed, which may result in the incomplete assessment of the mental status of participants. Furthermore, well-designed, cross cultural studies are needed to validate our findings.

## 5. Conclusions

The prevalence of PD is high in Chinese cancer patients. DT with a cutoff score of 4 is a comprehensive, appropriate, and practical initial screener for PD in heterogeneous cancer patients. Lower education levels, advanced cancer stage, active disease status, nondisclosure of diagnosis, lack of exercise, and emotional and physical problems are the predicting factors that should be considered together for effective management of PD in such population.

## Figures and Tables

**Table 1:** Summary of the Mainland China and Taiwan studies examining the validity of the distress thermometer (DT).

Author	Origin	Sample size	Cancer type	Setting	Reference criteria	DT cutoff score	AUC	SE	SP	PPV (%)	NPV (%)
Tang et al. [19]	Beijing	574	Mixed	Inpatient	HADS-T SCL-90	4 4	0.80 0.83	0.80 0.87	0.70 0.72		
Hong et al. [20]	Fujian	442	NPC survivors		HADS-T	4	0.72	0.42	0.85		
Deng et al. [21]	Sichuan	295	NPC		HADS-T	4	0.87	0.73	0.85	69.0	87.0
Wang et al. [12]	Sichuan	323	Lymphoma	Inpatient	HADS-T	5	0.91	0.75	0.86		
Zheng et al. [11]	Sichuan	172		Inpatient	HADS-T HADS-T	4 3	0.91 0.79	0.82 0.78	0.95 0.79		
Guan et al. [22]	Tianjin	441	Advance mixed cancer patients with pain	Inpatient	HADS-T	5	0.75	0.86	0.53	73.6	71.5

Wang et al. [23]	Taiwan	103	Mixed		DSM IV	4	0.89	0.98	0.73		
Chiou et al. [24]	Taiwan	786	Mixed	Inpatient	GHQ-12	4	0.79	0.72	0.80	29.2	96.2

NPC, nasopharyngeal cancer; HADS-T, Hospital Anxiety and Depression Scale-Total; SCL-90, Symptom Checklist-90; DSM IV, Diagnostic and Statistical Manual of Mental Disorders, 4th edition; GHQ-12, General Health Questionnaire-12; AUC, area under the curve; SE, sensitivity; SP, specificity; PPV, positive predictive value; NPV, negative predictive value.

**Table 2:** Bivariate and multivariate analysis of the association between the distress thermometer (DT) score and the sociodemographic, clinical characteristics of patients, and the problem list domains.

Demographic and clinical characteristics	No. of patients (%)	DT < 4(%)	DT ≥ 4(%)	Bivariate analysis *P* value	Multivariate analysis *P* value
Age (mean ± SD)	54.4 ± 11.2				

Source				**<0.001** ^*∗∗*^	0.07
Inpatients	784 (52.4)	328 (41.8)	456 (58.2)		
Outpatients	712 (47.6)	472 (66.3)	240 (33.7)		

Gender				0.17	
Male	729 (48.7)	403 (50.4)	326 (46.8)		
Female	767 (51.3)	397 (49.6)	370 (53.2)		

Marital status				0.51	
Married	1408 (94.1)	750 (53.3)	658 (46.7)		
Unmarried^a^	88 (5.9)	50 (56.8)	38 (43.2)		

Education level				**<0.001** ^*∗∗*^	**0.01** ^*∗*^
Junior high school or less	847 (56.6)	412 (48.6)	435 (51.4)		
More than junior high school	649 (43.4)	388 (59.8)	261 (40.2)		

Employment				**<0.001** ^*∗∗*^	0.48
Farmer	490 (32.8)	223 (45.5)	267 (54.5)		
Others	1006 (67.2)	577 (57.4)	429 (42.6)		

Monthly income (RMB)				**0.002** ^*∗*^	0.28
<3000	1062 (71.0)	522 (49.2)	540 (50.8)		
>3000	434 (29.0)	260 (59.9)	174 (40.1)		

Diagnosis				**0.02** ^*∗*^	**0.02** ^*∗*^
Known	1345 (89.9)	732 (54.4)	613 (45.6)		
Unknown	151 (10.1)	68 (45.0)	83 (55.0)		

Exercise				**<0.001** ^*∗∗*^	**<0.001** ^*∗∗*^
Lack (<150 min/week)	1010 (67.5)	441 (43.7)	569 (56.3)		
Often (>150 min/week)	486 (32.5)	359 (73.9)	127 (26.1)		

Disease status				**<0.001** ^*∗∗*^	**0.001** ^*∗*^
Complete remission	289 (19.3)	222 (76.8)	67 (23.2)		
Active disease	1207 (80.7)	578 (47.9)	629 (52.1)		

Type of cancer					
Lung	503 (33.6)	250 (49.7)	253 (50.3)	**0.03** ^*∗*^	0.85
Digestive	414 (27.7)	212 (51.2)	202 (48.8)	0.27	
Gynecological	163 (10.9)	104 (63.8)	59 (36.2)	**0.005** ^*∗*^	0.91
Breast	156 (10.4)	85 (54.5)	71 (45.5)	0.78	
Head and neck	107 (7.2)	55 (51.4)	52 (48.6)	0.65	
Urogenital	69 (4.6)	44 (63.8)	25 (36.2)	0.79	
Hematological	63 (4.2)	39 (61.9)	24 (38.1)	0.17	
Bone and soft tissue	21 (1.4)	11 (52.4)	10 (47.6)	0.91	

Stage of cancer				**<0.001** ^*∗∗*^	**<0.001** ^*∗∗*^
Advance	576 (38.5)	234 (40.6)	342 (59.4)		
Others^b^	920 (61.5)	566 (61.5)	354 (38.5)		

Treatment received				0.29	
Combined treatment^c^	834 (55.7)	436 (52.3)	398 (47.7)		
Single treatment	662 (44.3)	364 (55.0)	298 (45.0)		

Months since diagnosis				0.57	
<6 months	816 (54.5)	431 (52.8)	385 (47.2)		
>6 months	680 (45.5)	369 (54.3)	311 (45.7)		

Practical problem	894 (59.8)	374 (41.8)	520 (58.2)	**<0.001** ^*∗∗*^	0.12

Family problem	464 (31.0)	176 (37.9)	288 (62.1)	**<0.001** ^*∗∗*^	0.32

Emotional problem	1064 (71.1)	176 (37.9)	288 (62.1)	**<0.001** ^*∗∗*^	**<0.001** ^*∗∗*^

Physical problem	1174 (78.5)	503 (42.8)	671 (57.2)	**<0.001** ^*∗∗*^	**<0.001** ^*∗∗*^

DT (mean ± SD)	3.3 ± 2.6				

^a^Unmarried (single/divorced/separated/widowed). ^b^Other stages of cancer (stage I, II, III, and cancer stage under evaluation). ^c^Combined treatments (chemotherapy + surgery + radiotherapy + targeted treatment). ^*∗*^Bold values denote statistically significant values (*P* < 0.05). ^*∗∗*^Bold values denote statistically significant values (*P* ≤ 0.001).

**Table 3:** Sensitivity, specificity, Youden index, positive predictive value, negative predictive value, and clinical utility index of each distress thermometer (DT) cutoff score against the HADS-T scale.

DT cut off score	Sensitivity	Specificity	Youden index	PPV	NPV	UI+	UI-
Against HADS-T
0/1	0.95	0.40	0.35	0.61	0.90	0.58	0.36
1/2	0.93	0.52	0.45	0.66	0.88	0.61	0.46
2/3	0.89	0.72	0.61	0.76	0.87	0.68	0.63
**3/4** ^†^	**0.81**	**0.88**	**0.70**	**0.87**	**0.82**	**0.71**	**0.73**
4/5	0.60	0.92	0.53	0.89	0.70	0.54	0.65
5/6	0.34	0.96	0.30	0.90	0.59	0.31	0.57
6/7	0.22	0.98	0.20	0.93	0.55	0.20	0.55
7/8	0.13	0.99	0.12	0.94	0.53	0.12	0.52
8/9	0.05	0.99	0.56	0.95	0.51	0.05	0.51
9/10	0.03	0.99	0.03	0.96	0.50	0.03	0.50

HADS-T, Hospital Anxiety and Depression Scale-Total; PPV, positive predictive value; NPV, negative predictive value; positive utility index (UI+), sensitivity × PPV; negative utility index (UI−), specificity × NPV; Youden index, sensitivity + specificity-1. ^†^Bold values signify the balanced cutoff point with the highest Youden index.

**Table 4:** Risk factors for distress based on demographic, clinical variables, and problem list domains.

Variables	Odd ratio	95% confidence interval	*P* Value
Source of patients	1.29	0.972–1.732	0.07
Diagnosis known	0.64	0.435–0.951	**0.02** ^*∗*^
Lower education levels	1.39	1.060–1.825	**0.01** ^*∗*^
Less income	1.17	0.875–1.567	0.28
Active disease status	1.82	1.274–2.619	**0.001** ^*∗*^
Advanced cancer stage	1.85	1.424–2.405	**<0.001** ^*∗∗*^
Lung cancer	1.02	0.783–1.344	0.85
Gynecological cancer	0.97	0.630–1.514	0.91
Lack of exercise	3.03	2.307–3.989	**<0.001** ^*∗∗*^
Practical problems	1.25	0.941–1.684	0.12
Family problems	1.15	0.871–1.523	0.32
Emotional problems	3.54	2.540–4.942	**<0.001** ^*∗∗*^
Physical problems	8.62	5.468–13.594	**<0.001** ^*∗∗*^

^*∗*^Bold values denote statistically significant values (*P* ≤ 0.05). ^*∗∗*^Bold values denote statistically significant values (*P* ≤ 0.001).

## Data Availability

The data used to support this study are included within the article.
