# Corrigendum to “Performance of Distress Thermometer and Associated Factors of Psychological Distress among Chinese Cancer Patients”

**DOI:** 10.1155/2021/5318381

**Published:** 2021-02-09

**Authors:** Sudip Thapa, Huihui Sun, Ahmad Khalid Aalemi, Gaurab Pokhrel, Bangyan Wang, Sanuja Dahal, Shiying Yu

**Affiliations:** ^1^Cancer Center of Tongji Hospital, Tongji Medical College, Huazhong University of Science and Technology, Wuhan 430030, China; ^2^Department of Dermatology, Union Hospital, Tongji Medical College, Huazhong University of Science and Technology, Wuhan 430022, China; ^3^Department of Urology, Tongji Hospital, Tongji Medical College, Huazhong University of Science and Technology, Wuhan 430030, China; ^4^School of Nursing, Tongji Medical College, Huazhong University of Science and Technology, Wuhan 430030, China

In the article titled “Performance of Distress Thermometer and Associated Factors of Psychological Distress among Chinese Cancer Patients” [1], Dr. Ahmad Khalid Aalemi was missing from the authors' list. Dr. Aalemi shared his knowledge and experience during the study design, data cleaning, data analysis, and report writing. The corrected authors' list is shown above.
